# Contributions and challenges of community pharmacists during the COVID-19 pandemic: a qualitative study

**DOI:** 10.1186/s40545-022-00438-8

**Published:** 2022-06-16

**Authors:** Claire Durand, Eric Douriez, Aude Chappuis, Frédérique Poulain, Yazdan Yazdanpanah, Sylvie Lariven, François-Xavier Lescure, Nathan Peiffer-Smadja

**Affiliations:** 1 Infection Antimicrobials Modelling Evolution (IAME), UMR 1137, University of Paris, French Institute for Medical Research (INSERM), Paris, France; 2 Infectious Disease Department, University Hospital of Nice, Nice, France; 3 Union Régionale Des Professionnels de Santé Pharmaciens Ile-de-France, Paris, France; 4 Infectious Disease Department, Bichat-Claude Bernard Hospital, Assistance-Publique Hôpitaux de Paris, Paris, France; 5 National Institute for Health Research Health Protection Research Unit in Healthcare Associated Infections and Antimicrobial Resistance, Imperial College London, London, UK

**Keywords:** COVID-19, Infection prevention and control, Pandemic, Community pharmacy, Public health

## Abstract

**Background:**

Healthcare services across the world have been deeply impacted by the COVID-19 pandemic. In primary care, community pharmacists have had an important role in the frontline healthcare response to the pandemic.

**Objectives:**

This study aimed to explore the experiences, contributions and perceived challenges of community pharmacists regarding the provision of healthcare services during the COVID-19 pandemic.

**Methods:**

Semi-structured qualitative interviews were conducted with community pharmacists in France. Participants were recruited through a professional organization of pharmacists combined with a snowballing technique. Interviews were transcribed and then analyzed using thematic analysis.

**Results:**

A total of 16 community pharmacists participated in the interviews. Study participants described providing a range of novel services in response to the pandemic on top of continuing their usual services. All participants described providing preventative services to reduce and mitigate the spread of SARS-CoV-2, such as education on hygiene and social distancing, delivery of face masks and hand sanitizer and adjusting pharmacy premises. Most respondents also described being involved in SARS-CoV-2 detection through screening and performing antigen testing in pharmacies. Participants reported being actively involved in COVID-19 vaccination by educating the general public about vaccines, facilitating their distribution to general practitioners as well as administering vaccines. Over half the respondents described rapidly changing guidelines and service users’ anxiety as challenges to the provision of healthcare services during the pandemic.

**Conclusions:**

This study suggests that community pharmacists have significantly contributed to the response to the COVID-19 pandemic by ensuring continuity of pharmaceutical services and providing novel screening, testing and vaccination services. Their roles and responsibilities during the COVID-19 health crisis indicate that they can play an important role in the management of emerging infectious diseases.

**Supplementary Information:**

The online version contains supplementary material available at 10.1186/s40545-022-00438-8.

## Background

The coronavirus disease 2019 (COVID-19) outbreak was declared as a global pandemic by the World Health Organization (WHO) in March 2020 [1]. The spread of the COVID-19 pandemic has deeply impacted healthcare services across the world. Healthcare workers in both hospital and community settings have had to adapt and incorporate new healthcare services into their practice to tackle the spread of SARS-CoV-2 [2–4].

The role of pharmacists during public health crises such as pandemic outbreaks is not well-defined [5, 6]. Pharmacists’ clinical roles in previous health crises have not been widely recognized despite studies having demonstrated that the scope of pharmacy practice has expanded during disasters over the last 20 years and goes beyond logistics [5, 7]. The general lack of coordination between hospital and community healthcare poses a challenge to the integration of community pharmacists in health emergency response and their provision of clinical services during health crises. However, a Delphi study conducted in 2018 with an international expert panel of key stakeholders in disaster health and pharmacy identified 43 roles that pharmacists could be undertaking in disasters [6], including roles in all four phases of emergency management (i.e., prevention/mitigation, preparedness, recovery, response) [8]. Since the beginning of the COVID-19 outbreak, community pharmacists have had an important role in the frontline primary healthcare response to COVID-19 [4]. Indeed, pharmacists have remained widely accessible and available to the community and thus have often been the public’s first point of contact [9, 10]. Their role has been expanding over the course of the pandemic as they have gradually taken on the roles of educators, testers and vaccinators [11–13]. Despite pharmacists having provided new health services during the pandemic, little is known about how they implemented these services into their practice. There is also limited knowledge about the challenges and barriers that they have faced providing these novel public health services.

This qualitative study aimed to explore the scope of community pharmacists’ services during the COVID-19 pandemic as well as their views and experiences regarding the provision of these services. We sought to especially focus on the challenges that pharmacists have faced in their uptake of novel roles and responsibilities during the pandemic. In addition, we aimed to highlight lessons that can be learned from community pharmacists’ experiences during the COVID-19 pandemic to optimize their preparedness and response to future emerging infectious diseases and public health crises.

## Methods

### Study design

We conducted a qualitative study using semi-structured individual interviews. The study is reported in accordance with the Consolidated Criteria for Reporting Qualitative studies (COREQ) checklist [14] (see Additional file 1).

### Interview guide

We developed an interview guide, which was reviewed and validated by several infectious disease specialists and pharmacists. The interview guide consisted of thirteen questions about participants’ personal experiences and challenges in COVID-19 management and vaccination (see Additional file 2). The interview guide was checked for face validity during two preliminary individual interviews with community pharmacists, resulting in minor changes regarding the order of the guide’s questions.

### Sampling and data collection

The participant selection was informed by theoretical sampling. Participants had to be licensed pharmacists practicing in a community pharmacy in France to meet inclusion criteria. We recruited most of the respondents through a professional organization of community pharmacists, the “Union Régionale des Professionnels de Santé Pharmaciens Ile-de-France”, combined with a snowballing technique. We also used a convenience sampling method to recruit a few participants based on geographical proximity and availability.

An invitation letter was first sent to the “Union Régionale des Professionnels de Santé Pharmaciens Ile-de-France” who then sent it via email to their mailing list. A detailed information sheet and a consent form were then sent by email to pharmacists who responded to the initial invitation. A videoconference interview was arranged with pharmacists who consented to participate in the study. All interviews were conducted in French on the Zoom application by one researcher between February and May 2021. After written consent was obtained from the participants, the interviews were audio recorded using a dictaphone. Sampling and data collection were continued until theme saturation was reached. The socio-demographic data of each participant were collected at the end of each interview.

### Data analysis

Audio recordings were anonymized and transcribed verbatim using the automatic transcription software NVivo Transcription. Interview transcripts were then analyzed inductively by one researcher using qualitative data analysis software NVivo 12. Thematic analysis was carried out in the following steps: familiarization with the data to identify broad themes and categories, generating initial codes, categorization and classification of sub-themes and themes and analyzing the results through the framework analysis method [15]. The data analysis process was conducted in parallel with the interviews and in an iterative fashion. The results of the thematic analysis were reviewed and approved by the entire research team. A report was subsequently produced for which the themes and subthemes and quotes from the interviews were translated into English by two researchers. The open-source software R for Statistical Computing was used to analyze the participants' socio-demographic data.

### Ethics approval

This study was approved by the Institutional Review Board (IRB00011642) of the French Infectious Diseases Society (CER-MIT n°2021-0301).

## Results

### Sample characteristics

Sixteen community pharmacists participated in the interviews. The characteristics of the study participants are detailed in Table 1. Half the participants were women and mean age was 49 years. The majority of the respondents had more than 20 years of community pharmacy practice experience. Of all participants, 3 participants worked in rural areas and 13 in urban areas. Three pharmacists among the 16 participants practiced in low-income neighborhoods.

**Table 1:** Socio-demographic characteristics of participants

Socio-demographic characteristics	*n* = 16 (%)
Gender	
Male	8 (50)
Female	8 (50)
Age (years)	
< 30	3 (19)
30–39	1 (6)
40–49	3 (19)
50–59	8 (50)
≥ 60	1 (6)
Job title	
Pharmacy manager	12 (75)
Pharmacist staff	4 (25)
Community pharmacy practice experience (years)	
< 10	4 (25)
10–19	3 (19)
20–29	5 (31)
≥ 30	4 (25)
Number of pharmacists at the pharmacy (FTE)	
≤ 2	8 (50)
> 2	8 (50)
Number of pharmacy staff at the pharmacy (FTE)	
≤ 2	7 (44)
> 2	9 (56)
Pharmacy structure	
Group	11 (69)
Independent	5 (31)
Current practice location	
Neighborhood pharmacy	7 (44)
City center pharmacy	6 (37)
Rural pharmacy	3 (19)

*FTE* full-time equivalent

### Thematic analysis

Four main themes were identified through thematic analysis and are presented as follows. The first three themes referred to healthcare services provided by community pharmacists during the health crisis. These three themes and their subthemes are presented in Table 2.

**Table 2:** Community pharmacists’ novel services during the COVID-19 pandemic

General information and education	•First point of contact of service users for information •Educating service users on SARS-CoV-2 infection and symptoms •Educating service users on hygiene and social distancing measures •Main sources of information for pharmacists: health authorities, professional organizations, public media
COVID-19 clinical services	•Screening patients •Rapid SARS-CoV-2 antigen testing at the point of care •Reassuring patients •Informing patients about isolation measures •Delivering face masks •Providing monitoring instructions and informing about warning signs •Referring patients to GPs •Carrying out contact tracing
COVID-19 vaccination	•Educating service users on COVID-19 vaccines •Distributing vaccines to GPs •Administering vaccines

*GPs* general practitioners

#### Community pharmacists’ role in the prevention of SARS-CoV-2 transmission

“I can name so many things that we did. We adapted quite quickly. Above all, we were able to adapt pragmatically (…)” (P10).

Study participants reported community pharmacists have often been the public’s first point of contact for reliable COVID-19 information and explanations regarding government health measures. Many participants acknowledged that community pharmacies remaining open and accessible during lockdowns was an important factor in their role as educators. Pharmacists described providing various preventative health services to prevent and mitigate the spread of SARS-CoV-2, such as educating service users on hand hygiene and social distancing and supplying face masks and hand sanitizer.

In addition, pharmacists reported continuing and adapting their usual pharmaceutical services. Respondents reported being granted new responsibilities by health authorities, such as renewing and refilling prescriptions and providing certain medicines without medical advice. Many pharmacists described implementing a range of preventative measures and adjustments in pharmacies to continuously ensure care services and respond to the public’s needs in a safe environment. For instance, participants described extending opening hours, adapting pharmacy premises and adopting increased disinfection of premises and hand hygiene practices as well as strict interpersonal distancing. Study participants reported relying on several sources of information to update their knowledge and services, including newsletters from health authorities and online educational workshops by professional pharmacy organizations.

#### Community pharmacists’ role in SARS-CoV-2 detection and COVID-19 management

Respondents reported being actively involved in early detection of SARS-CoV-2. They described how their role evolved over the course of the pandemic. Indeed, participants reported that they initially screened patients and made appropriate referrals to general practitioners (GPs) and testing centers in suspected cases of COVID-19 before October 2020. The vast majority of participants described performing rapid antigen testing in pharmacies since October 2020.

“Sometimes, I feel like I've switched jobs because I have done so many things. Tests are very time-consuming and difficult to manage when things are in a hurry. Still, we managed to do it (…)” (P4).

In addition, some participants described managing patients who tested positive for SARS-CoV-2. For instance, they described reassuring and educating patients about COVID symptoms as well as providing clear information about isolation measures and warning signs. Participants described systematically referring patients at risk of severe COVID-19 to their GP for close follow-up. They also described conducting contact tracing. As for treatment, participants reported that they did not recommend patients any specific treatment other than antipyretics.

#### Community pharmacists’ role in COVID-19 vaccination

Many participants expressed actively contributing to COVID-19 vaccination by educating and encouraging service users to receive COVID-19 vaccines, facilitating the distribution of vaccines to GPs as well as administering vaccines in pharmacies from March 2021 onwards. Some respondents viewed their experience in influenza vaccination in two previous annual influenza vaccination campaigns as an enabler for their role in COVID-19 vaccination.

“So, I think that pharmacists have a role to play and besides they have the means to do it since we are trained to vaccinate against the flu.” (P2).

#### Perceived challenges of community pharmacists during the COVID-19 pandemic

The respondents described facing several challenges in the provision of healthcare services during the pandemic. These challenges are presented in Table 3. Over half the study participants described rapidly changing guidelines and service users’ COVID-19-related anxiety as major challenges to the provision of services during the pandemic. Some participants stated that pharmacists also struggled with non-compliance with isolation measures, mistrust of COVID-19 vaccines and misinformation about COVID-19 treatments and vaccines among service users. These participants felt that mistrust and misinformation likely negatively influenced patient self-management but could be helped with proper patient education. Three respondents also expressed a perceived lack of support and recognition for pharmacists from health authorities despite being essential frontline health workers. These pharmacists perceived a lack of support from health authorities in the fact that pharmacists were subjected to close quality inspections and retail price monitoring for face masks and hand sanitizers during the first wave of the pandemic in France.

“I think we had a lack of visibility (…) The difficulty is we had to readjust daily to measures that were different from the day before, even though we had already put everything in place (…)” (P7).

“People were afraid to consult their GP or to go to the hospital so the only close healthcare professional that they trusted was us. It's our job, but one of the big difficulties we have had is managing people's anxiety.” (P12).

**Table 3:** Perceived challenges of community pharmacists during the COVID-19 pandemic

Challenges	*n* = 16 (%)
Rapidly changing and conflicting guidelines	9 (56)
COVID-19-related anxiety among service users	9 (56)
Vaccine hesitancy and mistrust of COVID-19 vaccines among service users	6 (38)
Service users’ non-compliance with isolation measures	5 (31)
Decline in GPs' activity during the first wave of the pandemic	4 (25)
Misinformation about COVID-19 treatments among service users	4 (25)
Time and organization required for COVID-19 vaccination in pharmacies	4 (25)
Lack of support from health authorities	3 (19)
Shortage of vaccine doses	3 (19)

*GPs* general practitioners

## Discussion

The scope of community pharmacy practice appears to have progressively incorporated a range of novel public health services over the course of the pandemic. Community pharmacists have played an important role in the early detection and referral of COVID-19 cases, the implementation of preventative measures against SARS-CoV-2 transmission as well as in COVID-19 vaccination. Pharmacists’ novel services appear to have significantly contributed to the French national response to the pandemic by responding to the public's information and education needs and by facilitating the public’s access to testing and immunization services. Moreover, our results suggest that community pharmacists’ accessibility and skills facilitated the incorporation of these new services into their practice. Several participants expressed being motivated by a strong professional obligation to offer uninterrupted care services and to answer the public's needs and demands. Furthermore, one could argue that the pandemic has highlighted the potential of community pharmacists in public health emergencies and has raised the image of the pharmacy profession in the eyes of the public and health authorities.

The expanding role of community pharmacists in the management of infectious diseases has been an important research topic in recent years. Two systematic reviews focusing on community pharmacists’ practices in antibiotic prescribing and antibiotic stewardship suggested that pharmacists’ interventions were associated with increased quality of infection care, healthcare system benefits and high patient satisfaction [16, 17]. Thus, expanding pharmacists’ testing, infection management and vaccination services outside COVID-19 might benefit to patients and the primary healthcare system. One scoping review also acknowledged the uptake of novel public health roles by community pharmacists during the COVID-19 pandemic as well as the reinforcement of their roles in information and medication management [18]. The authors emphasized that the evolution of pharmacists’ roles during the health crisis could act as a catalyst for permanent professional change and mark the beginning of a new era for pharmacy practice. Another qualitative study exploring the experiences of pharmacists from 16 European countries during the COVID-19 pandemic also described that pharmacists have been faced with conflicting information in guidelines, patient fears of going to healthcare facilities, a lack of recognition by other healthcare professionals and a lack of coordination between community pharmacists and other providers [19]. There appears to be a lack of literature on the impact of community pharmacists' involvement in COVID-19 vaccination on the uptake of COVID-19 vaccination. However, one meta-analysis showed that pharmacists' involvement in influenza and pneumococcal vaccination, whether as educators, facilitators or vaccine administrators, resulted in increased vaccination rates [20]. Evaluations of the effects of community pharmacists’ participation in COVID-19 vaccination campaigns are needed.

However, several gaps and barriers to community pharmacists’ response were identified in this study, including rapidly changing guidelines and a lack of support from health authorities. These specific barriers could likely be improved by increased communication from health authorities and increased interprofessional collaboration between pharmacists and physicians. For instance, support for community pharmacists from expert physicians could be implemented through new communication channels, such as hospital hotlines to assist pharmacists in their COVID-19 clinical services. Other important challenges to pharmacists’ provision of health services during the pandemic were a lack of adherence to preventative measures and a mistrust of vaccines among service users. Increased collaboration between healthcare providers, with providers and public health institutions providing clear and similar messages to address users’ concerns, could help counter misinformation and increase user adherence to government measures. Given that community pharmacists are often the first point of contact between patients and the healthcare system in times of health crisis, patients could benefit from community pharmacists being more integrated into the organization of emergency health services.

One of this study’s strengths is the diversity of the participants’ socio-demographic status. This profile diversity allowed to capture the diversity of community pharmacists’ perspectives and experiences. Moreover, the study interviews were conducted during the third wave of the COVID-19 pandemic in France [21], thus reflecting pharmacists’ experiences and challenges during a high COVID-19 activity period. In addition, the interviews coincided with the implementation of COVID-19 vaccination services in community pharmacies [13], thus allowing to explore the perspectives of pharmacists on the provision of COVID-19 vaccination. However, this study focused solely on community pharmacists’ perspectives. Community pharmacists’ views and experiences are likely different from those of other primary healthcare providers. Studies focusing on the experiences of other healthcare providers and the perspectives of policy makers and service users are needed to fully comprehend the primary healthcare response to the pandemic.

## Conclusions

This study suggests that community pharmacists have significantly contributed to the collective response to the COVID-19 pandemic, by ensuring continuity of usual pharmaceutical services and incorporating novel public health roles as educators, screeners, testers and vaccinators into their practice. While providing these key public health services, community pharmacists have faced challenges in implementing official guidelines and in educating and managing service users. Community pharmacists’ roles and responsibilities during the COVID-19 pandemic suggest that they can play an important role in the management of emerging infectious diseases in primary care and should be integrated into the planning of health emergency responses.

## Supplementary Information

**Additional file 1. **COREQ checklist.

**Additional file 2. **Interview guide.

## Data Availability

The data sets used and/or analyzed during the current study are available from the corresponding author on reasonable request.

## References

[1] WHO Director-General’s opening remarks at the media briefing on COVID-19–11 March 2020. https://www.who.int/director-general/speeches/detail/who-director-general-s-opening-remarks-at-the-media-briefing-on-covid-19---11-march-2020. Accessed 24 Sep 2021.

[2] Perlini S, Canevari F, Cortesi S (2020). Emergency department and out-of-hospital emergency system (112-AREU 118) integrated response to Coronavirus Disease 2019 in a Northern Italy centre. Intern Emerg Med.

[3] Kerlin MP, Costa DK, Davis BS, Admon AJ, Vranas KC, Kahn JM (2021). Actions taken by US hospitals to prepare for increased demand for intensive care during the first wave of COVID-19: a national survey. Chest.

[4] Haldane V, Zhang Z, Abbas RF (2020). National primary care responses to COVID-19: a rapid review of the literature. BMJ Open.

[5] Watson KE, Van Haaften D, Horon K, Tsuyuki RT (2020). The evolution of pharmacists’ roles in disasters, from logistics to assessing and prescribing. Can Pharm J (Ott).

[6] Watson KE, Singleton JA, Tippett V, Nissen LM (2019). Defining pharmacists’ roles in disasters: A Delphi study. PLoS ONE.

[7] Miller S, Patel N, Vadala T, Abrons J, Cerulli J (2012). Defining the pharmacist role in the pandemic outbreak of novel H1N1 influenza. J Am Pharm Assoc.

[8] Wisner B, Adams J, World Health Organization (2002). , Environmental health in emergencies and disasters: a practical guide.

[9] International Pharmaceutical Federation. COVID-19 guidance: guidelines for pharmacists and the pharmacy workforce. 2020. https://www.fip.org/files/content/priority-areas/coronavirus/COVID-19-Guidelines-for-pharmacists-and-thepharmacy-workforce.pdf. Accessed 24 Sep 2021.

[10] Todd A, Copeland A, Husband A, Kasim A, Bambra C (2014). The positive pharmacy care law: an area-level analysis of the relationship between community pharmacy distribution, urbanity and social deprivation in England. BMJ Open.

[11] International Pharmaceutical Federation. COVID-19 guidance (Part 2): guidelines for pharmacists and the pharmacy workforce. 2020. https://www.fip.org/file/4729. Accessed 24 Sep 2021.

[12] Ministère des Solidarités et de la Santé. Arrêté du 26 octobre 2020 modifiant l’arrêté du 10 juillet 2020 prescrivant les mesures d’organisation et de fonctionnement du système de santé nécessaires pour faire face à l’épidémie de covid-19 dans le cadre de l’état d’urgence sanitaire. 2020. https://www.legifrance.gouv.fr/jorf/id/JORFTEXT000042469123. Accessed 24 Sep 2021.

[13] Ministère des Solidarités et de la Santé. Décret n° 2021–248 du 4 mars 2021 modifiant les décrets n° 2020–1262 du 16 octobre 2020 et n° 2020–1310 du 29 octobre 2020 prescrivant les mesures générales nécessaires pour faire face à l’épidémie de covid-19 dans le cadre de l’état d’urgence sanitaire - Légifrance. 2021. https://www.legifrance.gouv.fr/jorf/id/JORFTEXT000043216584. Accessed 24 Sep 2021.

[14] Tong A, Sainsbury P, Craig J (2007). Consolidated criteria for reporting qualitative research (COREQ): a 32-item checklist for interviews and focus groups. Int J Qual Health Care.

[15] Gale NK, Heath G, Cameron E, Rashid S, Redwood S (2013). Using the framework method for the analysis of qualitative data in multi-disciplinary health research. BMC Med Res Methodol.

[16] Saha SK, Hawes L, Mazza D (2019). Effectiveness of interventions involving pharmacists on antibiotic prescribing by general practitioners: a systematic review and meta-analysis. J Antimicrob Chemother.

[17] Wu JHC, Khalid F, Langford BJ (2021). Community pharmacist prescribing of antimicrobials: A systematic review from an antimicrobial stewardship perspective. Can Pharm J (Ott).

[18] Watson KE, Schindel TJ, Barsoum ME, Kung JY (2021). COVID the catalyst for evolving professional role identity? A scoping review of global pharmacists’ roles and services as a response to the COVID-19 pandemic. Pharmacy (Basel).

[19] Paudyal V, Cadogan C, Fialová D (2020). Provision of clinical pharmacy services during the COVID-19 pandemic: experiences of pharmacists from 16 European countries. Res Social Adm Pharm..

[20] Isenor JE, Edwards NT, Alia TA (2016). Impact of pharmacists as immunizers on vaccination rates: a systematic review and meta-analysis. Vaccine.

[21] Santé Publique France. COVID-19 : point épidémiologique du 4 mars 2021. https://www.santepubliquefrance.fr/maladies-et-traumatismes/maladies-et-infections-respiratoires/infection-acoronavirus/documents/bulletin-national/covid-19-point-epidemiologique-du-4-mars-2021. Accessed 24 Sep 2021.

