# Supplementary material for: Contributions and challenges of community pharmacists during the COVID-19 pandemic: a qualitative study
Source: J Pharm Policy Pract. 2022 Jun 16;15:43. doi: 10.1186/s40545-022-00438-8 (PMC9202330; doi:10.1186/s40545-022-00438-8)
Supplement: Supplementary file 2 — Additional file 2. Interview guide. [file 40545_2022_438_MOESM2_ESM.docx]

**Additional file 2: Interview guide**

Role and experiences

1. What do you think about the role of community pharmacists during the pandemic?
2. Since the beginning of the pandemic, what have been your sources of information on COVID-19?
3. What challenges have you faced in your pharmacy practice during the pandemic?

Management of COVID-19 cases

1. What are the symptoms or situations that make you think a patient might be positive for COVID-19?
2. What is your approach with confirmed cases of COVID-19?
3. In what situations do you refer COVID-positive patients to their GP or the emergency room?
4. What information or advice do you give to COVID-positive patients?
5. Do you recommend COVID-positive patients any specific medication?

COVID-19 vaccination

1. What role do you think pharmacists should play in COVID-19 vaccination?
2. What is your experience with influenza vaccination in the pharmacy?
3. What is your approach with users about COVID-19 vaccination?
4. What do you think are the benefits of administering COVID-19 vaccines in pharmacies?
5. What do you think are the barriers of administering COVID-19 vaccines in pharmacies?

Do you have any comments to add?
